# Editorial: Mechanobiology: Emerging Tools and Methods

**DOI:** 10.3389/fbioe.2020.00289

**Published:** 2020-04-09

**Authors:** Sara Baratchi, Khashayar Khoshmanesh, Charles David Cox, Guillermo Alberto Gomez

**Affiliations:** ^1^School of Health and Biomedical Sciences, RMIT University, Bundoora, VIC, Australia; ^2^School of Engineering, RMIT University, Melbourne, VIC, Australia; ^3^Molecular Cardiology and Biophysics Division, Victor Chang Cardiac Research Institute, Sydney, NSW, Australia; ^4^Centre for Cancer Biology, University of South Australia and SA Pathology, Adelaide, SA, Australia

**Keywords:** modeling, mechanobiology, molecular/cellular biology, biomaterials, bioengineering, micro/nanotechnologies

The effect of mechanical forces on tissue development was proposed more than one hundred years ago (Wolff, 1892; E. W. M, 1896; Thompson, 1992). During recent decades, with the advancement of tools and methods that allow us to mimic and measure mechanical forces, we have begun to understand how cells and tissues sense and respond to their physical surroundings. This understanding will provide fundamental knowledge on the role of mechanobiology in health and disease and new perspectives for the development of future therapies for their treatment.

Particularly, biomechanical forces play a crucial role in the development of different pathologies, as changes in physical forces of blood flow, shear stress, and pressure, play a significant role in the development of cardiovascular diseases (Baratchi et al., 2017). Likewise, tissue stiffness and solid stress as a result of abnormal tumor growth and resistance from the surrounding tissues affect tumor growth, metastasis, and treatment (Kalli and Stylianopoulos, 2018).

Mechanosensation and mechanotransduction are also key in the development and normal physiology (García-Añoveros and Corey, 1997; Lewin and Moshourab, 2004; Marshall and Lumpkin, 2012). For example, evolutionarily effective limb moment relies on limb biomechanics as well as mechanosensory feedback (Aiello et al., 2017), the sense of touch (Lumpkin et al., 2010) and ability to feel pain relies on the detection of mechanical stimuli by sensory neurons (Lewin and Moshourab, 2004) and cell fate, proliferation, differentiation, and death all rely on cell mechanics (Keller, 2012).

Therefore, we aimed this Research Topic to provide to the scientific community an overview of different techniques that have been developed to study the effect of mechanical forces on cells and tissues and provide an insight into different methods that have been developed to measure forces at the cellular and subcellular level.

The original research articles of this issue report the effect of aging on the viscosity of tendons fascicles and fibers (Karathanasopoulos and Ganghoffer) uniaxial dynamic loading on cultured microtissues (van Kelle et al.) and cyclic compressive stress on epithelial cells (Ho et al.). Method papers published in this issue include a novel microfluidic channel with integrated ridges to investigate the effect of disturbed flow on endothelial cells (Tovar-Lopez et al.) and a detailed protocol to adopt the Doppler echocardiography platform to analyse cardiac function (Benslimane et al.). Furthermore, Sianati et al. describes a protocol for investigating the response of mechanosensitive ion channels using pillar arrays and whole-cell patch-clamp techniques, and (Kriesi et al.) provides a technology report on an integrated flow chamber for *in vitro* quantification of mechanical stress using live-cell microscopy. In addition, this issue hosts comprehensive review articles on *in vitro* platforms to study the mechanobiology of cardiomyocytes (Chin et al.) and stretch technologies to investigate the mechanotransduction of cells in the cardiovascular system (Friedrich et al.). Furthermore, it features a comprehensive review of computation and experimental approaches to study abdominal aortic aneurysms (Salman et al.) and methods to investigate outside-in and inside-out mechanotransduction (Mohammed et al.).

Collectively, these articles provide a broad overview of different tools and methods that can be used to apply and study mechanical forces at cell and tissue levels, yet this is not a exhaustive list. The editors hope that the articles compiled in this issue will be worthwhile for researchers in the field of mechanobiology and that they will contribute to the advancement of knowledge.

Lastly, we would like to thank the efforts of all authors, co-authors, reviewers, and the Frontiers in Bioengineering and Biotechnology team who made this collection possible.

## Author Contributions

All authors listed have made a substantial, direct and intellectual contribution to the work, and approved it for publication.

### Conflict of Interest

The authors declare that the research was conducted in the absence of any commercial or financial relationships that could be construed as a potential conflict of interest.
